# Detecting One-Hundred-Year Environmental Changes in Western China Using Seven-Year Repeat Photography

**DOI:** 10.1371/journal.pone.0025008

**Published:** 2011-09-22

**Authors:** Huai Chen, Kaipu Yin, Haiyan Wang, Shenxian Zhong, Ning Wu, Fusun Shi, Dan Zhu, Qiuan Zhu, Weifeng Wang, Zhihai Ma, Xiuqin Fang, Weizhong Li, Pengxiang Zhao, Changhui Peng

**Affiliations:** 1 Laboratory for Ecological Forecasting and Global Change, College of Forestry, Northwest Agriculture and Forest University, Yanglin, China; 2 Chengdu Institute of Biology, Chinese Academy of Sciences, Chengdu, China; 3 Department of Biology Science, Institute of Environment Sciences, University of Quebec at Montreal, Montreal, Canada; University of Bristol, United Kingdom

## Abstract

Due to its diverse, wondrous plants and unique topography, Western China has drawn great attention from explorers and naturalists from the Western World. Among them, Ernest Henry Wilson (1876 –1930), known as ‘Chinese’ Wilson, travelled to Western China five times from 1899 to 1918. He took more than 1,000 photos during his travels. These valuable photos illustrated the natural and social environment of Western China a century ago. Since 1997, we had collected E.H. Wilson's old pictures, and then since 2004, along the expedition route of E.H. Wilson, we took 7 years to repeat photographing 250 of these old pictures. Comparing Wilson's photos with ours, we found an obvious warming trend over the 100 years, not only in specific areas but throughout the entire Western China. Such warming trend manifested in phenology changes, community shifts and melting snow in alpine mountains. In this study, we also noted remarkable vegetation changes. Out of 62 picture pairs were related to vegetation change, 39 indicated vegetation has changed to the better condition, 17 for degraded vegetation and six for no obvious change. Also in these photos at a century interval, we found not only rapid urbanization in Western China, but also the disappearance of traditional cultures. Through such comparisons, we should not only be amazed about the significant environmental changes through time in Western China, but also consider its implications for protecting environment while meeting the economic development beyond such changes.

## Introduction

**Figure 1:**
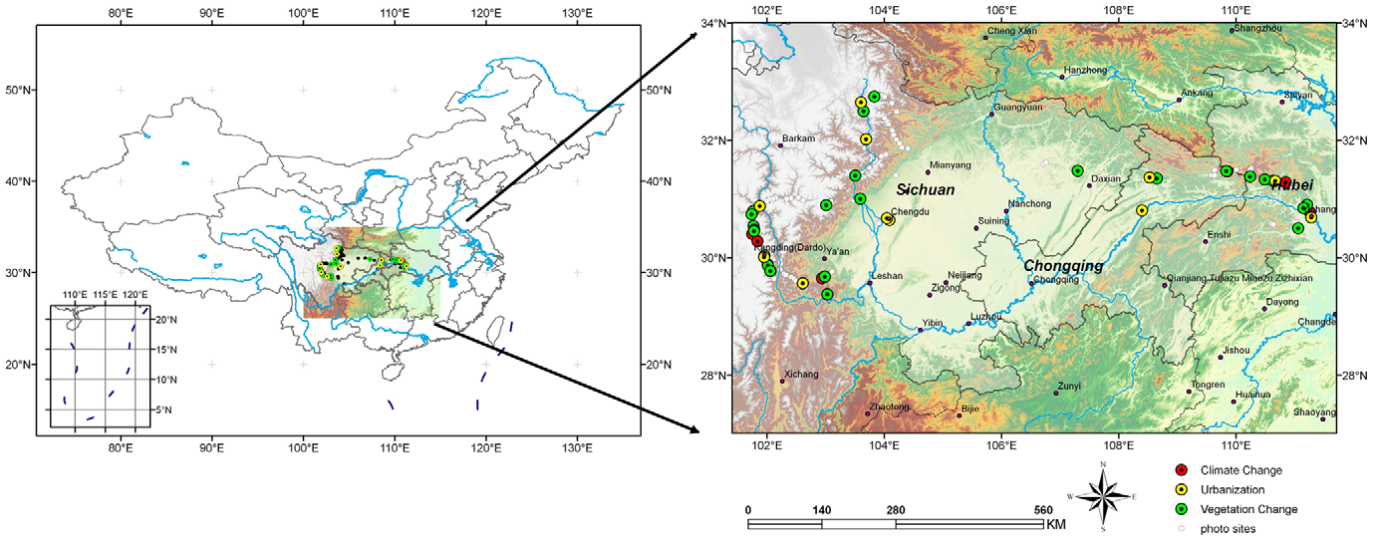
The study area and photo sites in Western China.

Western China is located in the transitional zone between the Southeast Qinghai-Tibetan Plateau and the Yangtze River Basin (Fig. 1), including Sichuan, Hubei provinces and Municipality of Chongqing. In the western alpine plateau and valley of Sichuan province, due to the continuous uplift of Qinghai-Tibetan Plateau, there are many specific animals and plants. Thus this region is named as the “cradle” for Chinese endemic animals and plants. In the mountains and river valleys of eastern Chongqing and western Hubei, because of barring of northward mountains, many ancient species survived during the Last Glacial Maximum, which made this region as a famous “refuge” for relict plants and animals. In recent years, As a significant reservoir of biodiversity highly threatened by human beings, Western China, especially mountains of Southwest, has been recognized as one of 34 biodiversity hotspots all over the world [1].

Due to its diverse, wondrous plants and unique topography, Western China, a remote region, has drawn great attention from explorers and naturalists from the Western World. However, it was not feasible for westerners to make expedition in Western China until the ending of Jiawu Sino-Japanese War (1894–1895), which made two southwestern cities, Chongqing and Wanxian, open to foreigners. Scientists and explorers including A. Henry, A.E.Pratt,P.Farges,E.H.Wilson,C.S. Scheneider came to Western China. Among them, Ernest Henry Wilson (1876–1930), known as ‘Chinese’ Wilson, travelled to Western China five times from 1899 to 1918. He not only collected about 65 thousand specimens of more than 4,700 kinds of plants, but also took more than 1,000 photos [2]. These valuable photos illustrated the society and environment of Western China a century ago.

Repeat photography is taking the same photo from the same spot with the same angle/lens at different times. It is usually used for monitoring and studying landscape or ecological change on the time scales of a century or less [3]. In United States and Europe such technique is widely used in research, while only a few studies used repeat photography to report vegetation change in China [4]. Moreover, these studies usually used repeat photography on a relative small scale to report landscape change [3], [4], [5], [6], climate change [7], [8], vegetation change [9], and fire ecology [10].

This present study had collected E.H. Wilson's old pictures since 1997, and then since 2004, along the expedition route of E.H. Wilson, we took 7 years to repeat photographing these old pictures. Comparing with Wilson's old pictures, we illustrated environmental change of Western China in the past hundred years on a region scale. However, in the last hundred years, due to great changes in social and economic aspects of China, including the increasing population and geological hazards in Western China, the environment of this region has undergone significant changes. This is one reason why we relocated and repeated only about one fourth (250) of 1,076 old pictures taken by Wilson in this region. In our study, through comparison between new and old pictures, we aimed to discuss climate change, vegetation change and urbanization in Western China through the past hundred years.

## Results

### Climate change

**Figure 2:**
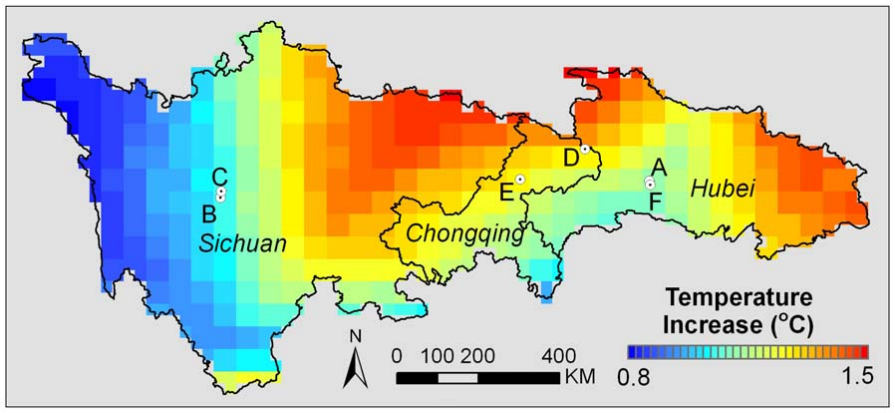
The Temperature increase though 100 years. The temperature data of the study area (Province Sichuan, Chongqing and Hubei) was extracted from CRUTS 3.0 climate datasets (http://badc.nerc.ac.uk/browse/badc/cru/data/cru_ts_3.00).The first decade mean temperatures of 20th and 21st centuries were calculated for the study area. The mean temperature increasing map was generated from the difference between these two periods (Mean temperature_2000–2006_ minus Mean temperature_1901–1910_).

**Figure 3:**
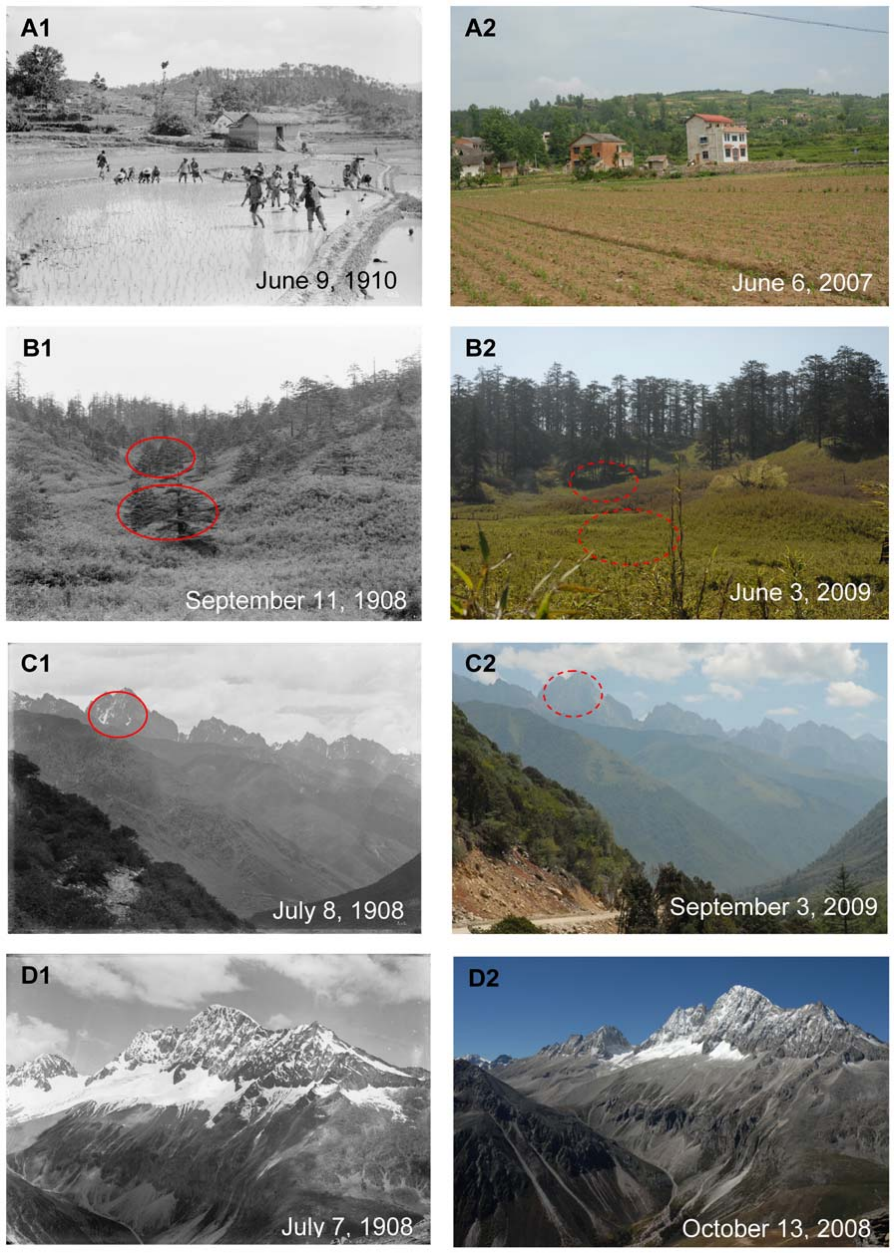
Evidence of climate change in Western China. **A:** Shicaoxi Village in Huangliang Township. Latitude and longitude: 31°17.246′N, 110°50.603′E. Elevation: 881 m. Note that people were transplanting rice in the historical photo. The paddy fields have been changed into dry lands. According to the planting season in 2007, the rice transplanting must be finished in early May in that area. **B:** Yuanyangchi in Hongya. Latitude and longitude: 29°38.958′N, 102°56.406′E. Elevation: 2693 m. Note that dense *Abies fabri* forests grew on the mountain slopes and bamboos (Fargesia) fully covered the understory in the historical photo. *A.fabri* trees have grown taller. Part of bamboo bushes have turned into grassland due to the decreased groundwater level because of climate change. **C:** Zhonggu Village in Kangding. Latitude and longitude: 30°16.941′N, 101°50.274′E. Elevation: 3213 m. Note that there were some snow-capped mountain peaks named Mt. Lotus because of the shape in the historical photo. However, nowadays these peaks have not been covered by snow, though the new photo taken two months later than the old one. **D:** Dapao Mountain in Kangding. Latitude and longitude: 30°24.359′N, 101°44.977′E. Elevation: 4530 m. Note that there was a snow-capped mountain, named Mt.Dapao in the historical photo. Obviously the snow coverage had withdrawn significantly due to climate change.

In Western China, climate change was obvious in the last century. The temperature increased by 0.8 to 1.5°C based on data from CRUTS 3.0 climate data sets (http://badc.nerc.ac.uk/browse/badc/cru/data/cru_ts_3.00) (Fig. 2). In agreement with other evidences in phenology change, community shifts and snow melting in alpine mountains, our research recorded the similar trend. In the historical photo of June 9, 1910, E. H. Wilson pictured people transplanting rice in Xinshan Xian in Hubei province (Fig. 3A1). Nowadays, due to reduction in spring precipitation, the paddy fields had changed into dry lands (Fig. 3A2). Moreover, according to the planting season in 2007, rice transplanting should be finished in early May in the same area, about one month earlier than that of 1910. Such advancing of rice planting was probably caused by the warming trend. On September 11, 1908, E.H.Wilson took a photo of dense *Abies fabri* forests grown on the mountain slopes in Sichuan with bamboos (*Fargesia*) fully covering the understory (Fig. 3B1). After one hundred years, *A.fabri* trees had grown taller (Fig. 3B2). But a great part of bamboo bushes had changed into grassland, probably due to the decreased groundwater level because of climate warming. There are many alpine mountains in Western China. Comparison between the old pictures and recent ones showed that almost all snow caps of mountains had retreated. Among them, significant melting of snow occurred in the peaks of Mt.Lianhua (Fig. 3C1) and Mt.Dapao (Fig. 3D1 and 3D2) . Moreover, the new photo showed the peaks of Mt. Lianhua had almost no snow in 2009, though it was taken in September, further in cold autumn than the old one, which was taken in July (Fig. 3C2).

### Vegetation change

**Figure 4:**
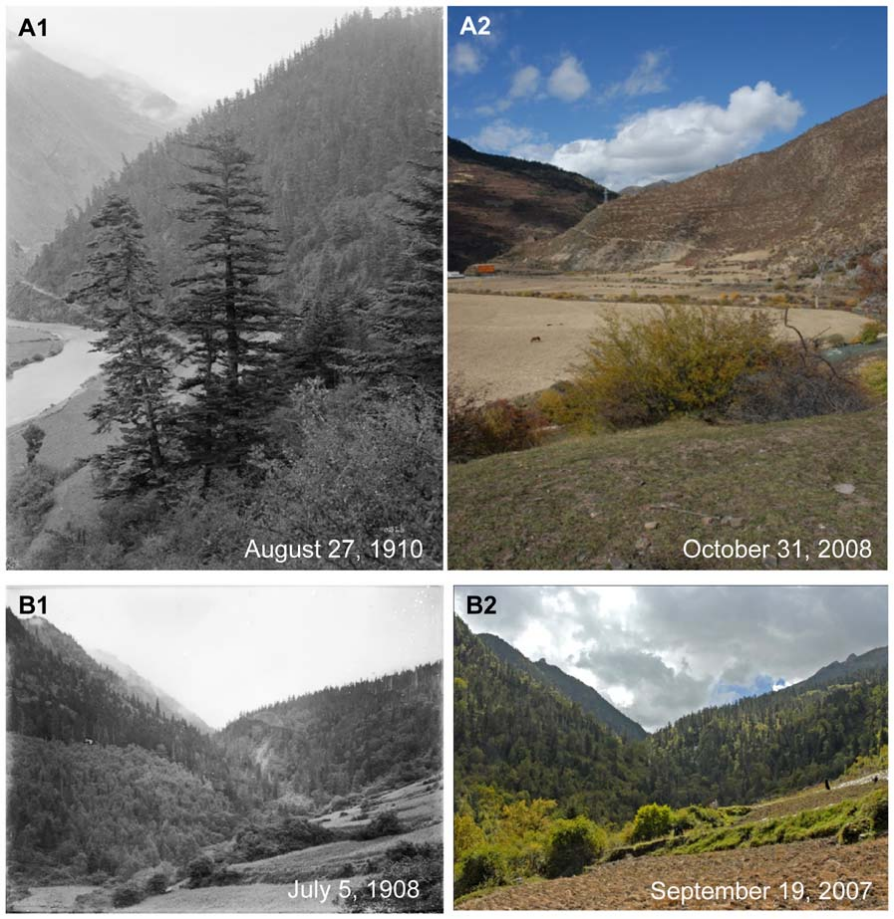
Changes of forests in Western China. **A:** Anhong Township. Latitude and longitude: 32°29.912′N, 103°38.720′E. Elevation: 2715 m. Note the riparian forest of the Minjiang River in Songpan County in the historical photo. After one hundred years, the forest had been totally cut down and replaced by bushes. In recent years, the area has been reforested, and some young *Abies faxoniana* trees have grown among the shrubs. **B:** Dakuiyong Village of Donggu Township. Latitude and longitude: 30°32.466′N, 101°46.324′E. Elevation: 3197 m. Note the thick forest close to Dakuiyong Village in Danba County in the historical photo. After one hundred years, the landscape had remained the same.

**Figure 5:**
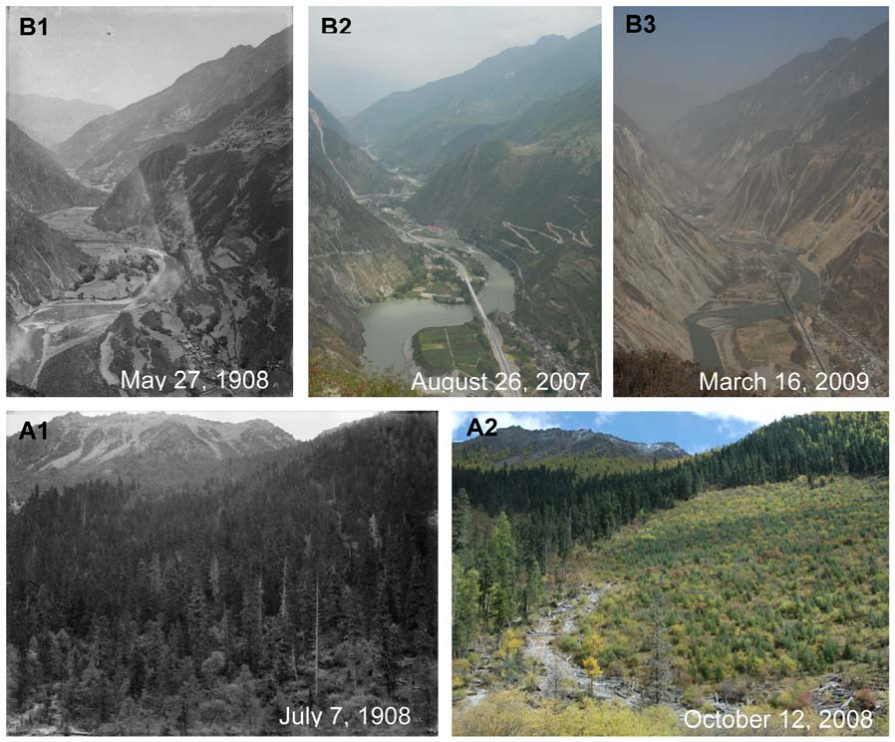
Changes of forests in Western China and earthquake dramatically destroyed the forest in Sichuan Province. **A:** Dakuiyong Village of Donggu Township. Latitude and longitude: 30°28.813′N, 101°47.092′E. Elevation: 3370 m. Note that a dense *Picea purpurea* forest in the upper Kuiyong river in Danba County in the historical photo. After one hundred years, part of the forest has been cut down more than 20 years. Now the regenerated seedlings are growing very well. **B:** Dankanliangzi of Mianchi Town. Latitude and longitude: 31°24.007′N, 103°30.439′E. Elevation: 1730 m. Note that a valley in the upper Minjiang River in the historical photo (B1). After one hundred years, the forests and bushes on the slopes have become denser (B2). The collapses and landslides caused by the Wenchuan Earthquake in 2008 have destroyed considerable parts of forests and farmlands on the mountain slopes (B3).

Western China is one of the regions of the most diverse and abundant vegetation of China due to its diverse topography and climatic condition. With the economic development in China, there are also significant changes of environment. Due to wars and economic development through the last hundred years, many forests have been cut or destroyed and the forest coverage has been significantly decreased in some parts of Western China, such as forests in Anhong Town (Fig. 4A1 and 4A2) and Dakuiyong Village of Donggu Town (Fig. 5A1 and 5A2), etc. Besides social and economic development, natural disasters are also a driver for vegetation change in Western China. Our photo repeat showed the damage by the natural disasters to forests. E.H.Wilson showed us a small gorge densely covered with forest of the earthquake zone in the historical photo (Fig. 6.1). After one hundred years, the forest has become denser (Fig. 6.2). But collapses and landslides caused by the Wenchuan Earthquake in 2008 destroyed some parts of forests on the mountain slopes (Fig. 6.3). In another historical photo, Wilson overlooked a valley in the upper Minjiang River (Fig. 5B1). After one hundred years, forests and bushes were denser in the valley, and the farmland had increased (Fig. 5B2). After the Wenchuan Earthquake, forests and farmlands had been dramatically destroyed by landslides (Fig. 5B3). Different from these examples, forests in some mountains of Tibetan communities have been remaining unchanged even through one hundred years (Fig. 4B1 and 4B2), probably because mountains are regarded as sacred and forbidden region in Tibetan culture.

**Figure 6:**
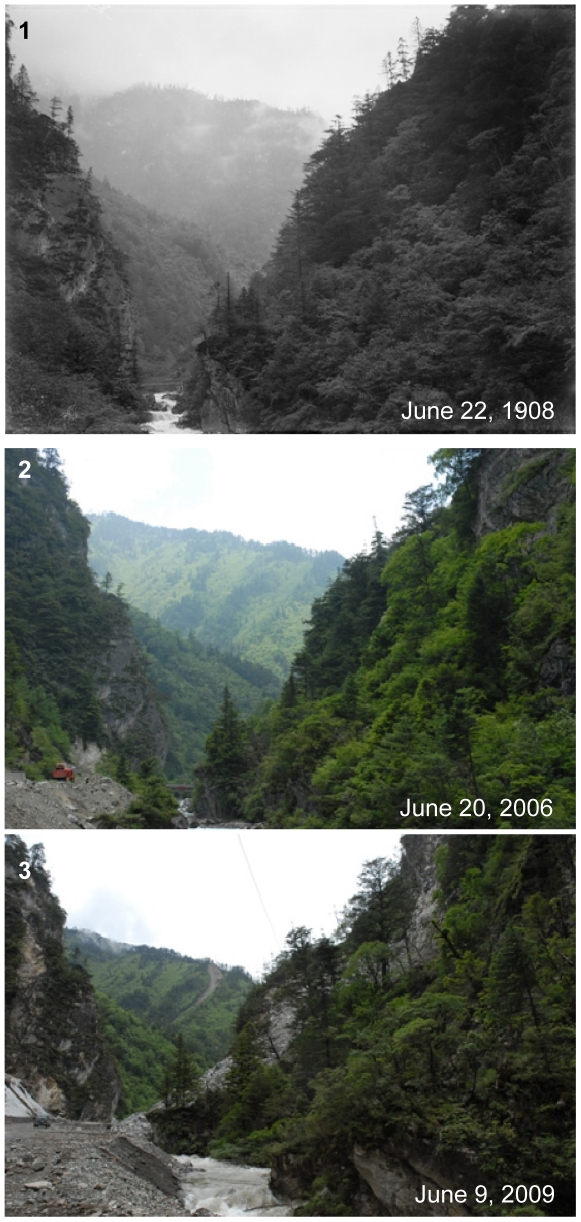
Sansheng Gorge in the Wolong Nature Reserve. Latitude and longitude: 30°53.793′N, 103°00.682′E. Elevation: 2550 m. Note the small gorge densely covered with forest in a valley in the historical photo (1). After one hundred years, the forest had become denser (2). The collapses and landslides caused by the Wenchuan Earthquake in 2008 destroyed some parts of forests on the mountain slopes (3).

**Figure 7:**
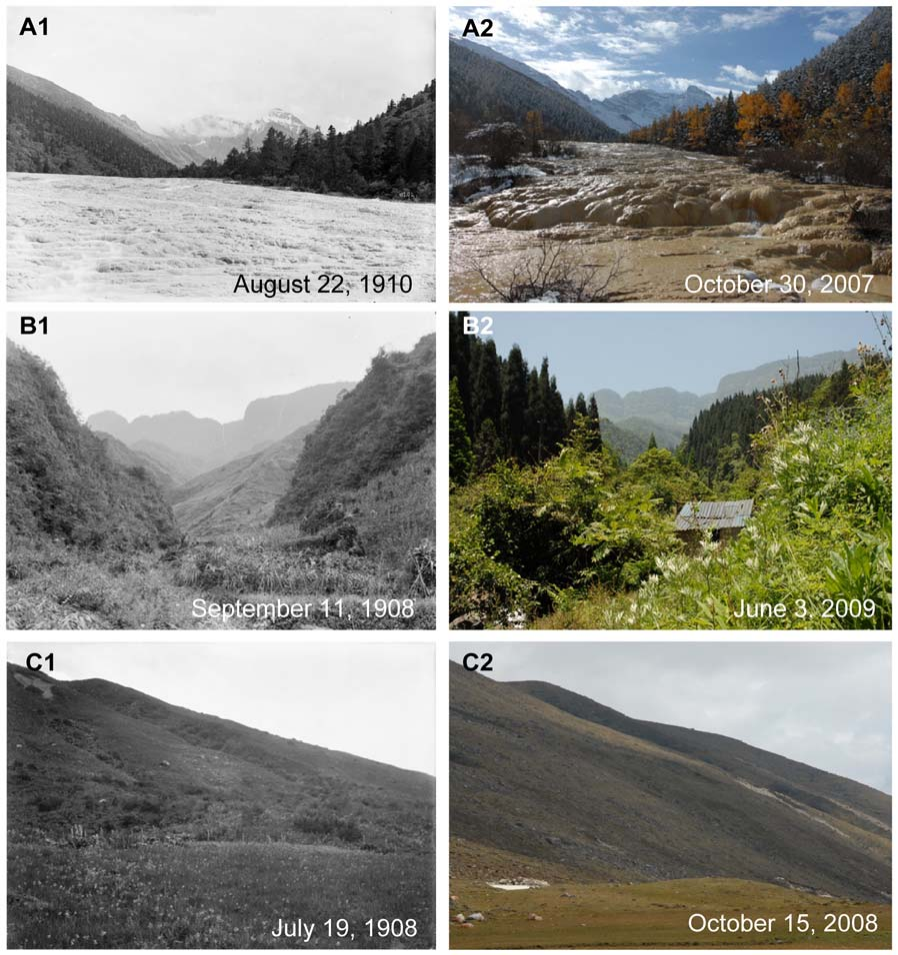
Unchanged and recovered forests, degraded grasslands in Western China. **A:** In Huanglong. Latitude and longitude: 32°44.742′N, 103°49.803′E. Elevation: 3197 m. Note that a calcified landscape with a dense coniferous forest in the historical photo. After one hundred years, the landscape has almost remained the same. **B:** By the Jinhua Bridge. Latitude and longitude: 29°40.766′N, 102°59.279′E. Elevation: 1148 m. Note that the almost flat top of Mt.Wawu with a piece of farmland on the right side in the historical photo. After one hundred years, the farmlands and the mountain slopes have been fully covered by tall forests of *Cryptomeria fortunei*. **C:** Daping Village of Xinxing Township. Latitude and longitude: 29°53.221′N, 102°00.967′E. Elevation: 3724 m. Note that an alpine grassland in south slope of Mt.Yajiageng in the historical photo. The grassland has degraded greatly and many places on the slope have become naked without vegetation due to overgrazing.

In our research, there were 62 pairs of pictures related to vegetation. Among them only 17 indicated that vegetation had degraded and 6 no significant change, but as many as 39 pairs indicated that vegetation had changed better. This is partly due to the development and implementation of China's unprecedented conservation actions, among which the Natural Forest Conservation Program (NFCP, initiated in 1998) and the Grain to Green Program (GTGP, initiated in 1999), are two of the biggest programs. The NFCP conserves natural forests through logging bans and afforestation, while the GTGP converts cropland on steep slopes to forest. Compared with the Wilson photos of mountain slopes in Western China one hundred years ago, natural forests were shown to be well protected due to NFCP (Fig. 7A1 and 7A2). Recent pictures also showed that most farmlands in the old ones had been changed to forest due to GTGP (Fig. 7B1, 7B2, 8A1 and 8A2).

**Figure 8:**
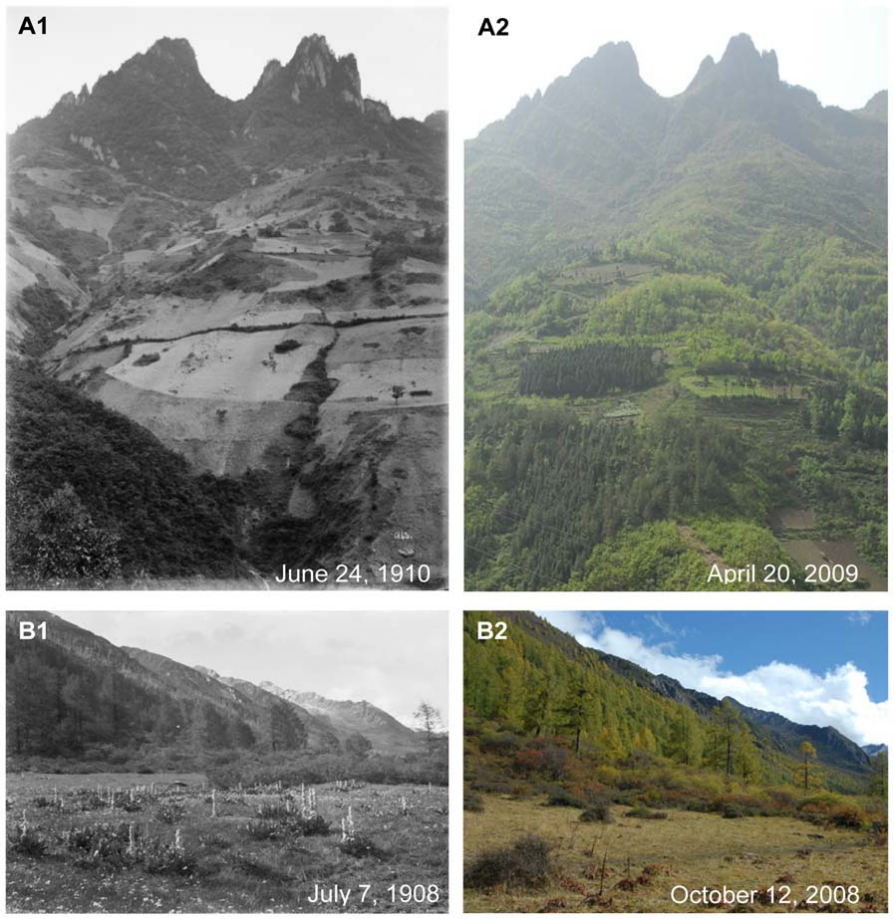
Recovered forests and degraded grassland in Western China. **A:** Zhangjiatang, a village in Shuangyang Township. Latitude and longitude: 31°28.621′N, 109°49.835′E. Elevation: 1070 m. Note the steep mountain slope with sparse vegetation and a large area of farmland without crops in the historical photo. After 100 years, the landscape was fully covered by dense forests with a scattering farmland under the implementation of the “the Grain to Green Program”. **B:** Near Dakuiyong Village of Donggu Township. Latitude and longitude: 30°27.340′N, 101°46.824′E. Elevation: 3918 m. Note the landscape with forest and grassland in Danba in the historical photo. The forest had not changed very much, while the grassland had degraded because of overgrazing. Grass height decreased significantly and there was an obvious reduction in Alexander Rhubarb (*Rheum alexandrae*).

In the last hundred years, great changes have not only taken in forest, but also in unique alpine meadows in Western China. In the July of 1908, Wilson took two photos of alpine meadows in Danba County and Luding County of Sichuan province (Fig. 8B1 and 7C1). At that time, the meadow was thick and diverse with flowering Alexander Rhubarb (*Rheum alexandrae*) and Sikkim Cowslip (*Primula sikkimensis*). Most of grasses were about 30 cm high. Now we relocated these two sites (Fig. 8B2 and 7C2). Comparing with Wilson's pictures, we found that two meadows had greatly degraded, regarding the reduction in plant height, vegetation cover (increasing sites without vegetation) and biodiversity (obvious decreasing in *R. alexandrae* and *P. sikkimensis*).

### Urbanization

**Figure 9:**
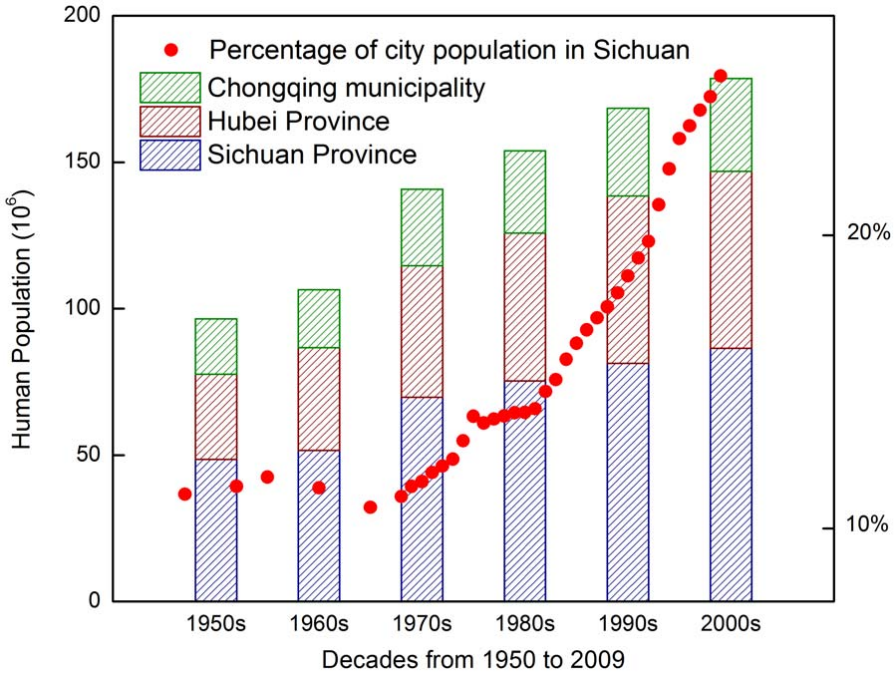
Decadal Changes of human populations from 1950 to 2009 in Sichuan Province, Chongqing municipality and Hubei Province.

**Figure 10:**
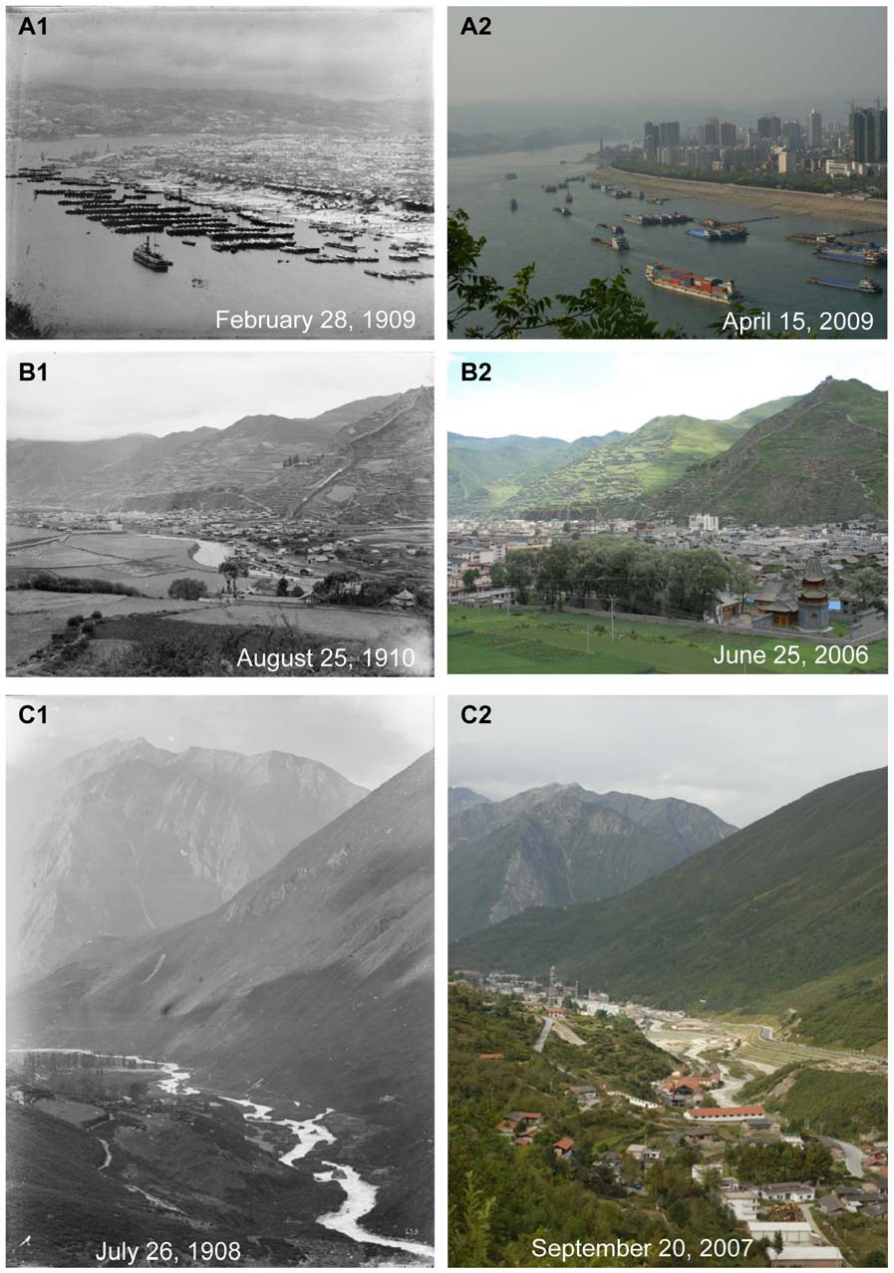
Urbanization of Yichang, Songpan and Kangding. **A:** Mount.Muji. Latitude and longitude: 30°41.148′N, 111°16.687′E. Elevation: 157 m. Note the port city of Yichang, with crowded one-storey houses, and the busy port in the historical photo. After 100 years, Yichang became a modern city with many tall buildings. **B:** Close to Songpan City. Latitude and longitude: 32°38.950′N, 103°36.221′E. Elevation: 2879 m. Note the old town of Songpan with Minjiang River flowing around in the historical photo. After one hundred years, the town had been largely extended. The small and low houses had been replaced by tall buildings, which also blocked the view of the Minjiang River. **C:** Zhaojiaping. Latitude and longitude: 30°00.585′N, 101°57.128′E. Elevation: 2810 m. Note the valley of the Zheduo River with a zigzagging flow and only two houses in the historical photo. After one hundred years, this peaceful place changed into the new town of Kangding City, whose tall and crowded buildings screened the Zheduo river.

**Figure 11 pone-0025008-g011:**
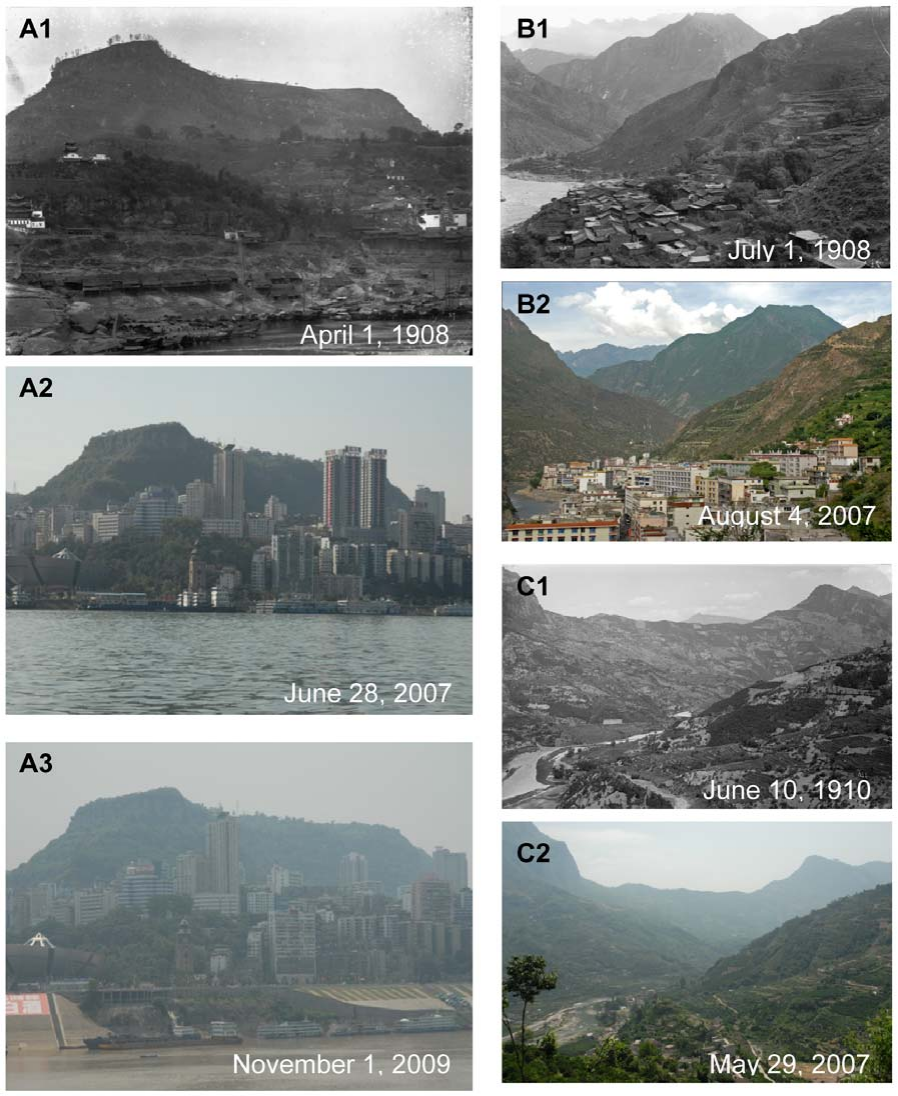
Urbanization of Wanzhou, Danba and Yuanquan. **A:** On the south bank of the Yangtze River. Latitude and longitude: 30°48.313′N, 108°23.666′E. Elevation: 160,162,172 m. Note that the view of Wanzhou city along the Yangtze River with small wooden boats in the river and some small, low houses in the historical photo (A1). After one hundred years, Wanzhou City has changed greatly with many new and tall buildings. Well-equipped riverboats have replaced with human-powered wooden boats (A2). It is also necessary to note that the lower part of the old city have been submerged due to the construction of the Three Gorges Dam (The water level has gone up by 50 m, A3). **B:** On the Minzhu Street in Danba Town. Latitude and longitude: 30°52.734′N, 101°52.760′E. Elevation: 1923 m. Note that the entire old town of Danba with less than 100 low and crowned houses in the historical photo. Due to the limited land on the bank of the Dadu River, although the town has not been bigger than the old one, many tall buildings have been erected. **C:** Yuanquan village in Nanyang town. Latitude and longitude: 31°18.632′N, 110°40.037′E. Elevation: 395 m. Note that the river valley with tiny terraced paddy fields full of water in the historical photo. Due to water shortage, the terraced paddy fields have become dry farmland. In the valley and on the lower slope, there have been many houses around.

One hundred years ago, Western China was regarded by the western world as a remote region with a mysterious veil. The well-known cities were ports or political centres, such as Chongqing, Yichang, Wanxian, Chengdu, etc. In the past 50 years, the population had greatly increased in Sichuan, Chongqing and Hubei (Fig. 9). Comparison of the pictures at a century interval revealed both high-speed urbanizations in Western China and the inherence and disappearance of traditional cultures. Port cities (Yichang and Wanxian) along the Yangtze River with small wooden boats in the river and some small, low houses in the historical photo had changed into modern cities with well-equipped riverboats and tall buildings (Fig. 10A1, 10A2, 11A1 and 11A2). Because of the construction of the Three Gorges Dam, the water level had risen to nearly 50 m, the lower part of cities along the Yangze river have been submerged (Fig. 11A3).

**Figure 12 pone-0025008-g012:**
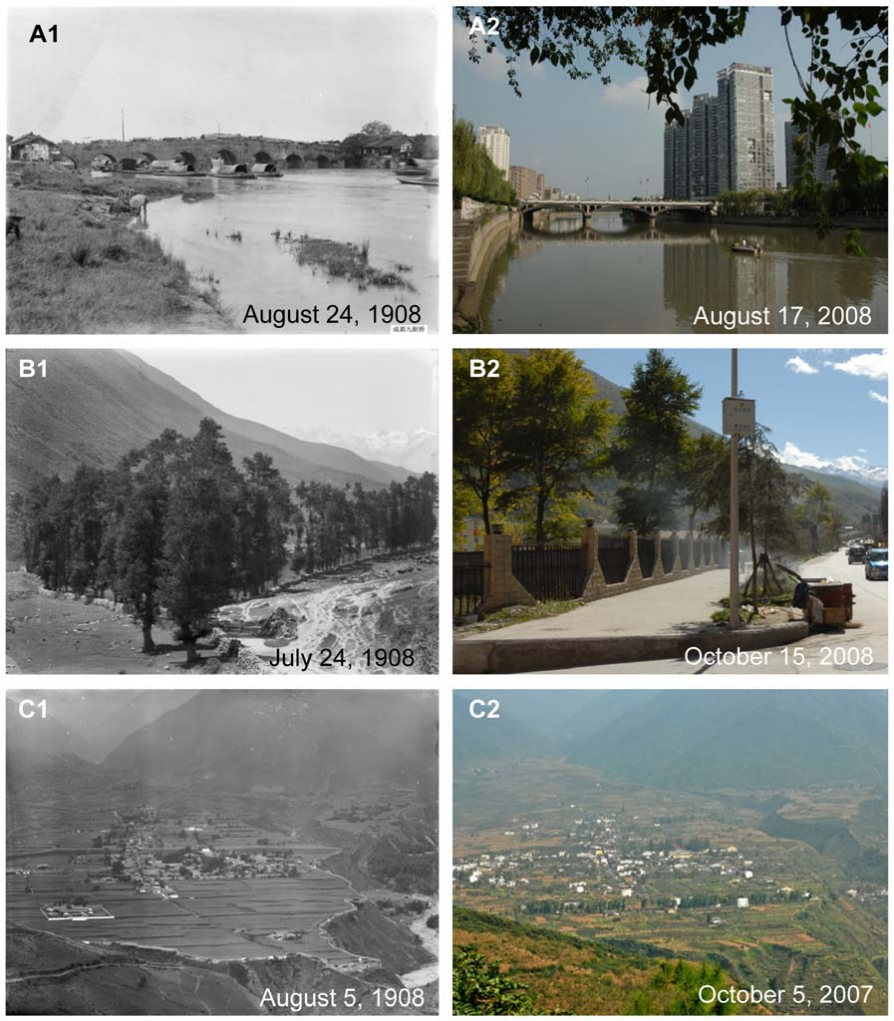
Urbanization of Chengdu, Kangding, Qingxi and its implications. **A:** By Jinjiang River in east Chengdu. Latitude and longitude: 30°38.624′N, 104°05.149′E. Elevation: 481 m. Note the river and a bridge with nine arches (“Marco Polo Bridge” called by Wilson) in the eastern part of Chengdu in the historical photo. After one hundred years, the view changed a lot. The old bridge was replaced by modern iron-and-cement bridge. The country view completely changed into a part of modern city with tall buildings on the river bank. **B:** Southern suburbs of Kangding. Latitude and longitude: 30°02.286′N, 101°57.584′E. Elevation: 2579 m. Note the Ancient Tea-Horse Road from Sichuan to Tibet in the historical photo. After one hundred years, this place became a part of Kongding City and the ancient road now a section of street in the city. **C:** Outside Qingxi Town. Latitude and longitude: 29°33.964′N, 102°36.623′E. Elevation: 1700 m. Note the peaceful view of the old Qingxi town with well-arranged houses and high walls in the historical photo. After one hundred years, the town had been largely extended in a disorganized way.

**Figure 13:**
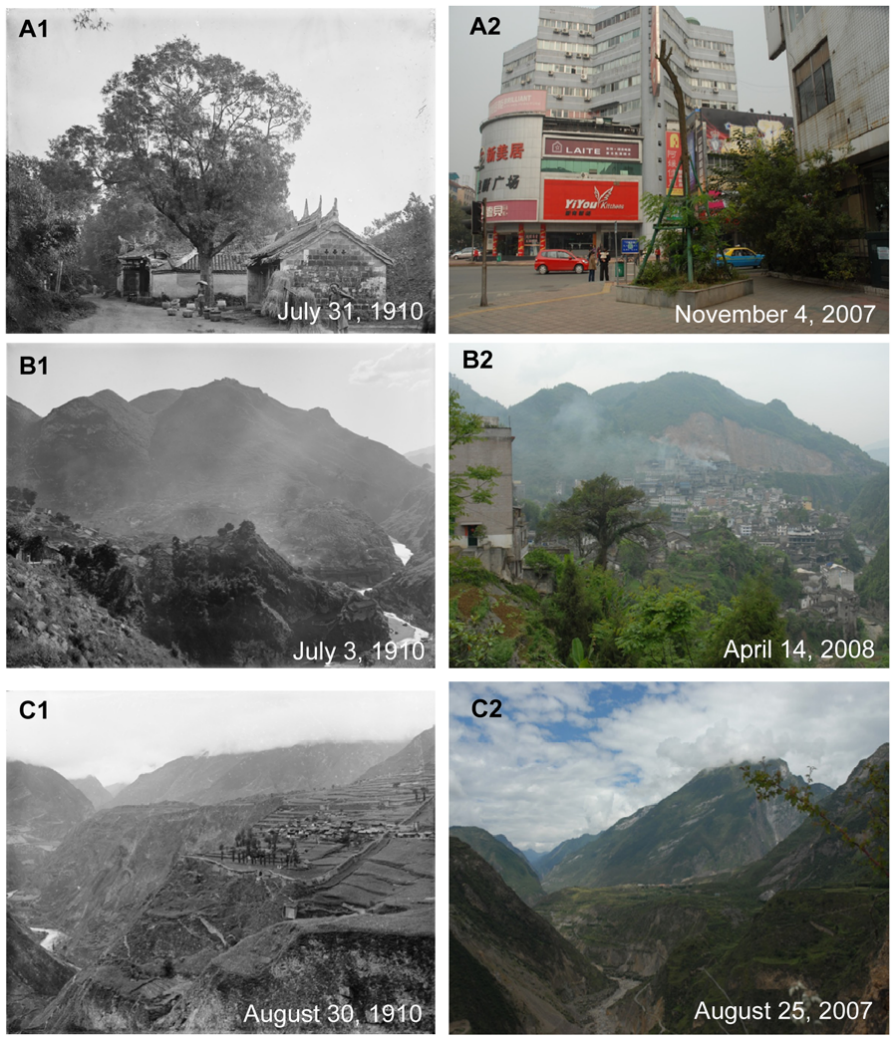
Extended urbanization of Chengdu, disordered urbanization of Wenquan, urbanization influenced by earthquakes and related landslides. **A:** On the Huaishu Street in Chengdu. Latitude and longitude: 30°40.488′N, 104°2.749′E. Elevation: 481 m. Note that a street with an old scholar tree (*Sophora japonica*) in west Chengdu in the historical photo. After one hundred years, this place has become a very busy street in the center of Chengdu City. The old tree might have died some years ago. Another tree of the same species is dying. **B:** Yanzi Street of Wenquan town. Latitude and longitude: 31°22.170′N, 108°31.411′E. Elevation: 706 m. Note that Wenquan town was prosperous with well-arranged builds in the historical photo. Many high buildings have been erected and trees and shrubs have been denser and bigger. Because of a cement factory, a large amount of limestone has been extracted and a large area of forests has been destroyed. **C:** Jiaochang Village in Diexi Town. Latitude and longitude: 32°01.160′N, 103°41.276′E. Elevation: 2356 m. Note that the old town of Diexi on a right tableland of the Minjiang River in the historical photo. After one hundred years, this town has been destroyed by a strong 7.5-magnitude earthquake in 1933. After that, the town has been moved to Jiaochangba.

Through the one hundred years, landscapes inside city had also changed greatly. As a result many old photos taken in cities could never be reshot. Taking Chengdu for example, due to extended urbanization, some places in the suburb had changed into an important part of the city (Fig. 12A1 and 12A2) and an old street with a few houses became a busy street in the center of the city (Fig. 13A1 and 13A2). Not only well-known cities of Western China had undergone high-speed urbanization, but also smaller cities, even towns and villages in mountainous regions of Southwest China. As the important connection between Han and Tibetan nationalities, the old town of Songpan county in western remote region of Sichuan deeply impressed Wilson with its beauty and cultural significance one hundred years ago (Fig. 10B1). Another Tibetan county, Danba at that time had less than 100 low and crowned houses in the town (Fig. 11B1). But now, these cities had largely extended, and most small, low houses had been replaced by tall buildings (Fig. 10B2 and 11B2). Also due to city extension, one part of the famous ancient Tea-Horse Road from Sichuan to Tibet near Kangding now became a street in the town of Kangding (Fig. 12B1 and 12B2). Moreover, peaceful valleys with no or just a few settlements had changed into a prosperous village or even the town of a county with tall and crowded buildings (Fig. 10C1, 10C2, 11C1 and 11C2). However, in the progress of urbanization, some old towns with well arranged houses had become a “cement forest” in a disorganized way (Fig. 12C1, 12C2, 13B1 and 13B2). In Western China, earthquakes and related secondary landslides had also influenced the progress of urbanization. Diexi earthquake (M*_L_* = 7.5) in the August of 1933 almost destroyed the whole town by lowering the town by 50 meters. Now on the same location, we could find any footprint of the old town of Diexi photographed by Wilson a hundred years ago (Fig. 13C1 and 13C2).

## Discussion

At the same sites of photographing of one hundred year interval, great differences were shown in Wilson's lens and ours. Such differences abounded in climate change, vegetation change and urbanization. Climate change, especially climate warming, is unequivocal, amply evidenced by increasing of global average air and ocean temperatures, widespread melting of snow and ice and rising global average sea level [11]. In Western China, the trend of climate warming was also obvious (Fig. 2 and Fig. 3). Under the global warming, spring events at temperate latitudes have been advanced by 2.5 days per decade since 1971 [12]. This is consistent with our study, where we found rice transplanted a month earlier than that in 1910. Besides phenology, climate change can also alter community composition in alpine forests of Western China [13]. In a warming world, less winter snow and the melting of snow pack decreases the snow cover in the caps of mountains [14], [15] . Our repeat photos showed the same trend in Western China. In terms of climatic change, comparison of Wilson's photos with ours indicated a trend of warming not only in a specific spot or aspect but in many aspects and the whole area of Western China from western Hubei province to western Sichuan province.

In the history, dense and diverse forests were widely distributed in Sichuan province in this region [16]. Due to the religious reason, some mountains are regarded as sacred in Tibetan communities of Western China [17]. Without human disturbance, forests in these mountains did not show obvious change through the hundred years (Fig. 7A1 and 7A2). However, because of increasing population, reclaim for agriculture, and wars, forests had changed a lot. This is the reason why out of 62 picture pairs related with vegetation 17 photos indicated degraded vegetation 6 photos no significant change. Besides the pictures, some data also evidenced such degradation. For example, the forest coverage was 40% in Sichuan province in the early 20 century; in 1940s, the figure decreased to 19%; in 1950s, it further deceased to only 9%; and in 1980s it increased to 13% [2].

Western China is one of the most seismic regions in China. According to Sichuan Earthquake Administration, there have been 175 strong earthquakes (Richer magnitude *M_L_*>5.0) since 1900 in western Sichuan. Therefore, earthquake and its secondary landslides have destroyed many forests in Western China. The Wenchuan Earthquake (May 12, 2008, M*_L_* = 8.0) disturbed a serene mountainous area of about 20,000 km^2^ along the fault zone in the Longmen Mountains of Sichuan Province. After the strong earthquake, forest cover in Sichuan Province decreased by 0.5%, which means 330,000 ha (equivalent to about half the area of Singapore) of dense natural and planted forests were destroyed [18]. In the epicentre, more than 10% of forests was destroyed by the earthquake [19].

Our research observed the dramatic change of the forest in Western China (Fig. 6.1, 6.2, 6.3, 7B1 and 7B2). Since 1998, Chinese government implemented two unprecedented conservation programs, NFCP and GTGP. After the implement of such two programs, forest cover in Sichuan province has increased to 30% with a rate of 1% per year and 20 million ha natural forests have been effectively conserved since 1998 [2]. Even after the Wenchuan earthquake, these two conservation programs have been regarded to offset some of the combined effects of human disturbance and earthquake-induced landslides on this region's forest cover [19]. This is probably the reason why photo comparison (39 out of 62 photo pairs related with vegetation) showed a change to the better of the vegetation through the past century.

In meadow ecosystem, long-term overgrazing has resulted in considerable deterioration and even desertification in Western China [20]. Though a very large grassland conservation program (Known as “Grain to Green Program”) was initiated in 2003 by central government of China, recovery of alpine meadows in Western China to its condition one hundred years ago still needs more time and care. Moreover, a recent program, known as “New Tent-Dwelling Life” project, launched by the government of Sichuan Province will improve nomads' living conditions and indirectly reduce the grazing on the alpine meadow [21].

In the past one hundred years, the population of Western China has increased greatly since the early twentieth century, e.g. the population of Sichuan province (including Chongqing, which was set as municipality of China in 1997) has increased from 49.9 million [22] to 120.6 million [23], [24]. Wide spread urbanization takes place not only in well-known cities but also in remote regions in Western China (Fig. 10–13). Taking Sichuan for example, during the late 1950s, only 11% of Sichuan's population lived in cities, but this rate rose to 26% by 2009 (Fig. 9). Especially since the late 1970s, China has been undergoing a period of economic reformation and rapid urbanization, with the contribution of cities to gross national product (GNP) increasing from 69.9% to 85.9% [25]. On the other hand, such rapid urbanization has also led to serious environmental and ecological consequences, including more polluted air and water [26], local climate change [27], reduction in vegetation cover and biodiversity [25].

Through comparisons with new and old photos at a century interval, we should not only be amazed about the significant environmental changes and economical development in Western China, but also pay great attention to the relation between economic development and environment protection, and consider measures to balance between environment protection and economic development. One century ago, Western China was poor and close, with only slight conflict between environment and development. Maybe, at that time, “poverty” was not “the greatest polluter to the environment”. Because of few or even no pervasive human influence, environment had been inconsciently protected except for some religious reasons in this region [17]. However, though at present there is unavoidable conflict between economic development and environment protection [28], national and international policy has usually ignored the environment [29]. Such proposition is often described as the “inverted-U” shaped relation between per capita income and some environmental quality (with increasing per capita income, environment first degrades, then improves after the turning point). In fact, the inverted-U relation has only happened in some cases [29].Therefore, to get a win-win balance between environment and economy, regional sustainable development should simultaneously combine economic and environmental perspectives [30]. Now the protection paradigm has been shifted to a positive orientation aimed to reduce the environmental risks, ameliorate degraded vegetation and disorganized urbanization, and improve the livelihoods of local people through such an open and industrialized century of China [31]. In fact, two of the protection programs (NFCP and GTGP) have gained success in both aspects of ecology and socioeconomics [32]. Though human actions have a dominant impact on ecosystems [33], natural disturbances such as disasters (earthquakes and landslides) and climate change have also greatly shaped the Western China. However, effective protection can lessen the damage of natural disasters. For example, though the Wenchuan earthquake greatly destroyed a part of a peaceful region in Western China, Viña et al. (2010) pointed out that NFCP and GTGP have reduced its damage to some extent. The historically ecological changes in the past century of Western China inform us that sustainable development is possible in such a human-dominated vulnerable region, though there are many great challenges to approach the balance of development and protection.

## Materials and Methods

### Site description

Our study region covered a vast area of Southwest China (N28°40′∼33°10′ and E 101°30′∼110°30′, a.s.l. 60 m∼7556 m) about 300,000 km^2^, which included Sichuan Province, Chongqing Municipality, and the western Hubei Province (Fig. 1). This region is the transitional zone from mountainous area of eastern China to the Tibetan Plateau. From east to west, there are many high mountains, like Shennongjia, Dabashan, Longmenshan , Mingshan, Qionglai, Daxueshan, etc. Meanwhile, this region is also a transitional zone from subtropical to temperate. From south to north, there are several large rivers, including Yangtze River, Jinsha River, Dadu River, Min River, Jianglin River, Han River, and so on. Due to its variable climate and complicated topography, there are about 120,000 kinds of plants and more than 1,000 kinds of animals, including some precious “live fossil” plant such as *Davidia involucrata*, *Ginkgo biloba*, *Cathaya argyrophylla*, *Pseudolarix amabilis* and world-famous rare animals like Giant panda, golden monkey and Chinese sturgeon.

### Old photo selection and collection

Most of the photos about Western China in early 20^th^ century were taken by foreign ambassadors, adventurers and preachers. Among all, a comparatively comprehensive view of Western China was recorded by the old pictures taken by Ernest Henry Wilson (1876–1930), a noted English plant collector. His photos were chosen as our source of re-shooting and comparison due to the following two reasons: 1) no large time intervals. About 90% of his photos were taken in 1908 and 1910; 2) wide coverage. His photos covered almost all aspects of ecosystems and main cities in Western China.

We took several years (1997 to 2003) to collect Wilson's photos of Western China mainly from his publications (books and papers, etc.), news media (like newspapers) in old days, Arnold Arboretum of Harvard University (where Wilson worked) and related websites. Finally, we collected 1,056 pictures taken by Wilson from 1907 to 1911. For all the origin photos used in this paper we have got the permission of authors and publishers. We also collected Wilson's journals and found the detailed description for each photo we used.

### Photograph repeat

After collecting Wilson's photos and preliminary locating their origin, we spent several years (2004-2009) to relocate and take the same shot at the very viewpoint of each old photo. Meanwhile we recorded all related information about altitude, longitude, elevation and the shooting date. Altogether we repeated photographing 250 old photos. We chose to compare Wilson's old photos with our new ones to find the changes through about 100 years, and selected 4 pairs, 28 pairs and 12 pairs to illustrate climate change, vegetation change and urbanization in Western China, respectively.
